# Dynamic Plasmonic Coupling in Gold Nanosphere Oligomers: Mechanically Tuned Red and Blue Shifts for SERS/SEF

**DOI:** 10.3390/bios15030181

**Published:** 2025-03-13

**Authors:** István Tóth, Cosmin Farcău

**Affiliations:** 1 National Institute for Research and Development of Isotopic and Molecular Technologies, 67-103 Donat, 400293 Cluj-Napoca, Romania; 2 Institute for Interdisciplinary Research in Bio-Nano-Sciences, Babes-Bolyai University, 42 T. Laurian, 400271 Cluj-Napoca, Romania

**Keywords:** plasmonic oligomers, dynamic tuning, molecular sensing

## Abstract

Controlling the surface plasmon resonances of metal nanostructures is crucial for advancing numerous high-sensitivity optical (bio)sensing applications. Furthermore, dynamically adjusting these resonances enables real-time tuning of the spectrum of enhanced electromagnetic fields in the near field, thereby regulating the optical interactions between molecules and the metal surface. In this study, we investigate the plasmonic behavior of linear oligomers composed of gold nanospheres using finite-difference time-domain electromagnetic simulations. The extinction spectra of linear arrangements such as dimers, trimers, and quadrumers are obtained for different sphere sizes, interparticle gaps, and polarization of the incident light. In view of (bio)sensing applications based on plasmon-enhanced optical spectroscopy such as surface-enhanced Raman/fluorescence (SERS/SEF), the sensitivity of various coupled plasmon modes to the variation of the interparticle gap is evaluated. The achievement of both red-shifting and blue-shifting plasmon modes offers ways to mechanically control the optical response of the linear oligomers in real-time and design new optical sensing protocols. Based on these findings, both an approach for trapping molecules into SERS hotspots and an approach for dual-mode SERS/SEF using a single excitation wavelength are proposed, contributing to the future development of (bio)sensing protocols.

## 1. Introduction

Noble metal nanoparticles and nanostructured films, due to their unique interaction with light, specifically through surface plasmons (localized or propagative), have garnered significant interest in the field of nanophotonics and nanotechnology [1,2]. Localized surface plasmons (LSP), the collective oscillations of free conduction electrons inside the metal nanoparticle embedded in a dielectric medium, allow for spatially and spectrally selective interaction with light, yielding tunable enhanced electromagnetic fields, absorption, or scattering. These effects have paved the way for a myriad of applications in diverse fields such as (bio)medical, chemical and biosensing [3,4,5], catalysis [6,7], photovoltaics [8], and many others, and are also leading to groundbreaking, fundamentally relevant findings in these fields. The basis for sensing applications is the high sensitivity to changes in the local environment, such as refractive index variations or the presence of molecules near the metal surface [9]. The utilization of plasmonic nanoparticles for biosensing and environmental monitoring has revolutionized the detection of biomolecules and analytes with unparalleled sensitivity and specificity. Furthermore, surface-enhanced Raman spectroscopy (SERS) has flourished as a powerful analytical tool, enabling the detection of trace molecules through the enhancement of Raman signals by several orders of magnitude [10,11,12]. When fluorophores interact with the strong electromagnetic field generated by surface plasmons, the excitation rate and radiative decay of the fluorophores increase, resulting in enhanced fluorescence signals [13]. Surface plasmon-assisted fluorescence enhancement, or surface-enhanced fluorescence (SEF), has thus also been utilized in various applications, including (bio)sensing [14,15]. The advancements of SERS and SEF have opened up new possibilities for highly sensitive and selective detection techniques, with potential applications in fields such as diagnostics, environmental monitoring, and biomedical research.

The optical response of noble metal nanoparticles varies significantly depending on their individual or collective behavior [16,17]. Single nanoparticles exhibit distinct optical properties, whereas the interaction between coupled nanoparticles, such as dimers, results in enhanced and tunable optical responses. Understanding the transition from individual to coupled nanoparticles in plasmonic oligomers of various geometries is pivotal in unraveling the full potential of these structures in optical applications [18]. Real-time control of interparticle distances presents a compelling avenue for tailoring the optical response of nanoparticle assemblies [19,20,21]. This dynamic modulation offers unprecedented opportunities for biosensing applications using optical spectroscopy, including the emerging field of dual SERS-SEF sensing [22,23,24]. Moreover, the active manipulation of interparticle distances holds promise for the development of strain and deformation sensors with exceptional sensitivity and versatility [25].

In this work, we investigate in detail the optical response of linear plasmonic oligomers composed of gold nanospheres supported by a stretchable elastomeric substrate. By using finite-difference time-domain electromagnetic simulations, the extinction spectra of different-sized linear arrangements (dimers, trimers, quadrumers) are obtained for various gold sphere sizes (20, 100, 250 nm), a wide range of interparticle gaps (3–500 nm), and polarization of the incident light. The sensitivity of various coupled plasmon modes to substrate strain (relative elongation, achieved by variation of interparticle gap) is then evaluated. We then analyze the spatial distribution of the electric fields near the plasmonic oligomers. The stability of the coupled modes to size and gap variability is also investigated. Based on our findings, both an approach for trapping molecules into SERS hotspots, and an approach for dual-mode SERS/SEF using a single excitation wavelength are proposed as contributing to the development of future (bio)sensing advanced methodologies.

## 2. Method

Finite-difference time-domain (FDTD) simulations of the interaction between the electromagnetic radiation and linear oligomers of gold nanospheres were performed using the Ansys Lumerical 2022 FDTD software (Canonsburg, PA USA). We constructed several simulations with linearly arranged (along the *x*-axis of the simulation box) dimers, trimers, or quadrumers of gold nanospheres placed on a dielectric substrate. In every case, we studied the interaction with plane-wave radiation for different gaps between the nanoparticles, ranging from 3 to 500 nm. The plasmonic response was evaluated by calculating the extinction (combined scattering and absorption) cross-section using the total-field-scattered field method. The spectral grid of the radiation source was set between 400 and 1600 nm, with a step of 2 nm. We also investigated the plasmonic response in terms of the polarization of the incoming radiation, that is parallel or perpendicular to the axis of the different oligomer chains. The size of the nanoparticles was also varied, performing simulations for small (with a radius *R* = 10 nm), medium (*R* = 50 nm), and large nanospheres (*R* = 125 nm). The refractive index of the medium in which the simulations were performed was 1, while that of the substrate was 1.43, to resemble polydimethylsiloxane (PDMS). The material properties of the gold nanoparticles were used by Johnson and Christy. The mesh accuracy factor of the simulation area was set to 6. We also used a refined mesh with a resolution of 1 nm inside the total field-scattered field box in order to capture the details of the electric field variations even at small gap values between the nanoparticles. Including an additional smaller mesh in the region between the particles was also tested. Results showed only minor differences, so we decided that the 1 nm mesh was satisfactory. Please see the Supplementary Materials Figure S1 for a comparison between 1 nm mesh over the entire particles and a 1 nm mesh over the particles with a supplementary 0.2 nm mesh in the gap region for the case of 4 spheres with *R* = 50 nm and *d* = 3 nm, *x* polarization.

## 3. Results and Discussion

**Figure 1:**
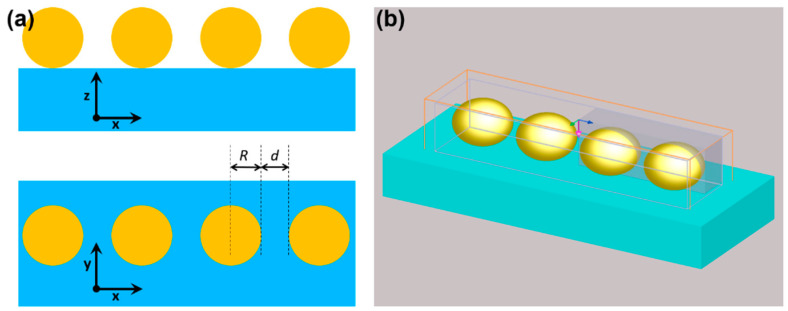
(**a**) Schematic description of the analyzed oligomers, composed of spheres of radius *R*, spaced by distance (gap) *d*, and placed on a dielectric substrate; (**b**) perspective view of an actual simulation setup, with the simulation volume (brown rectangular box) and light source propagation direction (purple arrow) and polarization (blue arrow).

Finite-difference time-domain (FDTD) simulations were used to evaluate the extinction cross-sections of various-sized linear nanoparticle oligomers. The arrangement of the gold nanosphere oligomers under study is depicted in Figure 1: spheres of radius *R*, separated by interparticle gap *d* are aligned along the *X* direction on a dielectric substrate. A 3D rendering showing four nanospheres inside the simulation volume and together with the excitation source, based on the total field-scattered field method, is also presented in Figure 1b. Linear oligomers composed of two, three, or four gold nanospheres, sized 20 nm, 100 nm, or 250 nm are arranged on a dielectric substrate mimicking the elastomeric material PDMS. By obtaining their optical response for different interparticle distances, we are modeling the behavior of such plasmonic systems upon stretching of the PDMS substrate.

**Figure 2:**
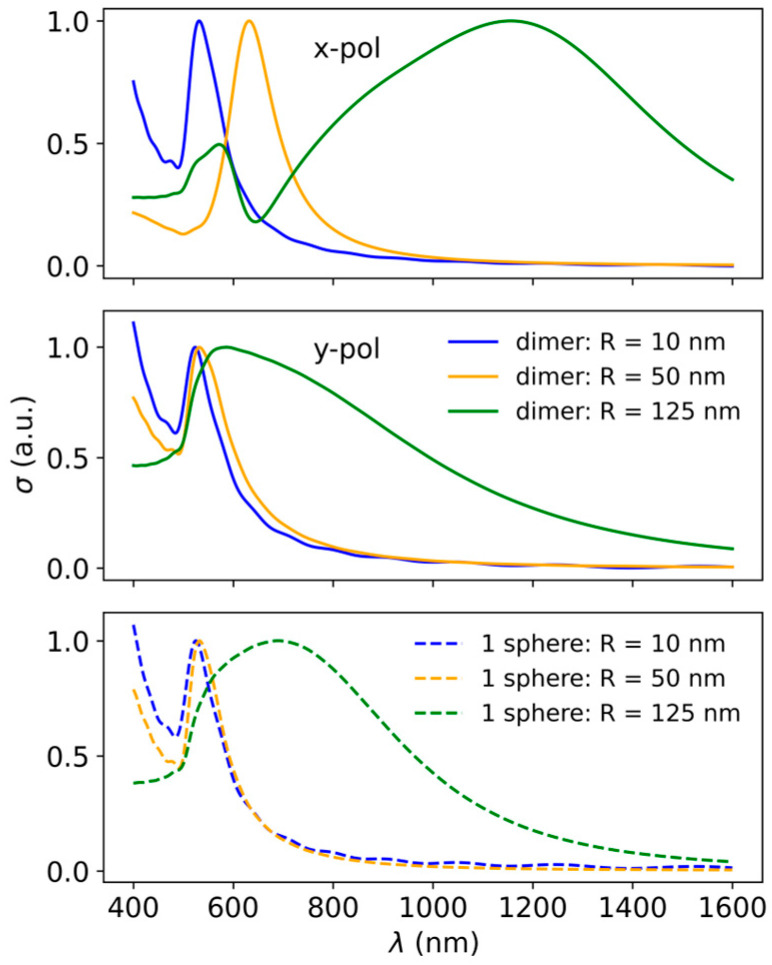
Extinction spectra for sphere dimers (solid line) and single spheres (dashed line) (sphere radius *R* = 10, 50, and 125 nm) at a fixed distance (*d* = 3 nm), for the *x* and *y* polarizations of the incident light.

To highlight the importance of particle size, interparticle distances, and light polarization in such multiparticle systems, we present in Figure 2 the extinction spectra of single spheres (dash lines) with *R* = 10, 50, 125 nm, and their dimers (solid lines) with 3 nm interparticle gap. First, note that while the single-particle spectra for smaller spheres exhibit narrow resonances, corresponding mainly to dipolar excitations, that for the largest sphere (*R* = 125 nm) are very broad and asymmetric, comprising contributions from quadrupole and octupole excitation at short wavelengths, and dipole excitation at longer wavelengths. In large spheres, the plasmon resonance is also broadened due to increased damping effects caused by interactions with the surrounding environment, or dynamic depolarization, which also leads to a red-shift and broadening [26]. As can be observed in the case of dimers, when light is polarized along the dimer axis, a shift of the plasmon resonance relative to the corresponding single particle spectrum is observed and indicates an interaction between the plasmons on the two particles. For the smallest spheres, this shift is small, and it increases drastically for larger spheres, taking values of 8/100/466 nm for *R* = 10/50/125 nm. At the same time, the dimer spectrum becomes much broader for the largest spheres.

### 3.1. Effect of Particle Size and Interparticle Gap on Plasmon Resonances

**Figure 3:**
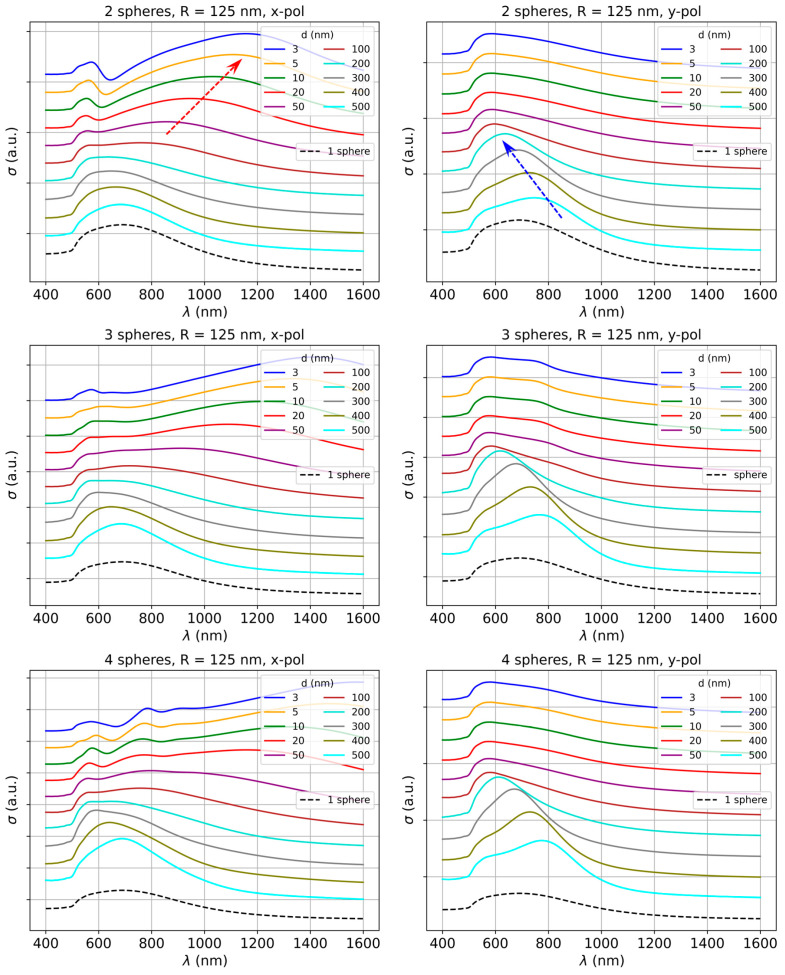
Extinction spectra for dimers, trimers, and quadrumers composed of gold nanospheres (*R* = 125 nm) as a function of interparticle distance (*d* = 3 to 500 nm), for the *x* and *y* polarizations of the incident light. Spectra for single spheres are also included. The spectra were shifted along the vertical direction for better visualization. Arrows indicate the shift of the resonance maximum.

As it was illustrated above for the dimer case, the particle size, interparticle distance, and incident light polarization all have an important impact on the plasmonic response of the gold nanosphere dimers. We further analyzed in detail the effects of all these parameters for different-sized linear plasmonic oligomers, namely dimers, trimers, and quadrumers. We present here the results for the largest spheres considered (*R* = 125 nm). Figure 3 shows the extinction spectra of dimers, trimers, and quadrumers of gold nanospheres, for different gap values, as shown in the legends. The cross-section for a single nanosphere is also plotted on the figure, as reference. The top row of Figure 3 shows the spectra for a dimer system, where a two-peak structure may be observed in the case of *x* polarization: a dipole mode at longer wavelengths, while the one at shorter wavelengths corresponds to an emerging quadrupole mode. The dipole mode peak is red-shifted (moves towards longer wavelengths) as the gap decreases, while the quadrupole peak is essentially not affected by these gap changes. We mention here that for smaller spheres (*R* = 10 nm and *R* = 50 nm) only a single peak is observed (in Figures S2 and S3 of the Supplementary Materials), which is also red-shifting for decreasing gaps. The shift is a measure of the plasmonic coupling strength among the spheres, which becomes stronger for larger spheres. For *y*-polarized radiation at small, few nm interparticle distances, we have almost identical extinction spectra, but with a different structure compared to the spectra of the smaller particles, a very broad and asymmetric band towards the IR side. Meanwhile, at larger gap values, a different coupling is signaled by the blue-shift (toward shorter wavelengths) of the peak in the range 800–600 nm as the gap decreases from 500 nm to 100 nm. A similar kind of blue-shift has been observed before in nanoparticle dimers excited by light polarized perpendicular to the interparticle axis and attributed to the enhancement of the restoring forces for the oscillating electrons due to the presence of the charge distribution of the neighboring particle [27,28]. However, it has not been discussed very often in the literature, and even less for trimers and quadrumers or exploited for sensing.

The results for linearly arranged trimers are presented in the middle row of Figure 3. The overall behavior of the extinction spectra is similar to the dimer case; however, the red-shift of the dipole peak for *x* polarization is more pronounced than for dimers. There is also a slight shift observable in the case of the quadrupole peak for decreasing gap values starting around a gap of 100 nm. Again, for *y* polarization, we essentially do not observe any change regarding the position of the peaks, except for the blue-shift in the range 800–600 nm as we decrease the gap from 500 to 100 nm. The bottom row of the figure shows the results for a linear quadrumer system. The red-shift of the dipole peak is the largest for this configuration compared to dimers and trimers for *x*-polarized irradiation. Further, the spectra are similar to the trimer case, except for a hint of an additional peak emerging at sub-10 nm gaps between the spheres. In *y* polarization, the spectra are very similar to the trimer case. Regarding the polarized response analyzed here, it could be argued that in many applications unpolarized light is used for excitation. The unpolarized response can be estimated by averaging between the results of x and y-polarized excitations in our results. Such unpolarized spectra are included in the Supplementary Materials, Figure S4. However, when devising new applications and new protocols, if polarization control can offer some advantages, this is not difficult to implement, as it only involves a polarizer filter positioned on the excitation beam.

**Figure 4:**
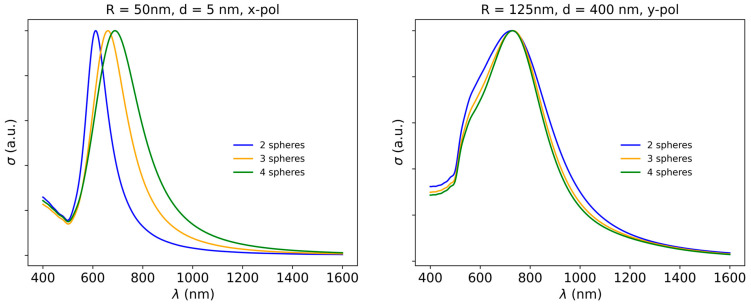
Dependence of the extinction spectrum on the number of spheres in the linear oligomer for: (**left**) *R* = 50 nm, *d* = 5 nm, *x* polarization; (**right**) *R* = 125 nm, *d* = 400 nm, *y* polarization.

The dependence of plasmon resonance behavior on the number of particles forming the linear oligomers is also worthy of a short discussion, especially to emphasize once more the differences between the blue- and red-shifting coupled plasmon resonances. As examples, two distinct cases are presented in Figure 4: oligomers composed of spheres with *R* = 50 nm separated by *d* = 5 nm under *x* polarization, and oligomers composed of spheres with *R* = 125 nm separated by *d* = 400 nm under *y* polarization.

In the first case, the particle number has a significant impact on the spectral profile, the coupled plasmon resonance red-shifting from 612 nm to 660 nm, and to 688 nm, when moving from the dimer to the trimer, and quadrumer, respectively. In the second case, the blue-shifting resonance in the large sphere oligomers is only slightly sensitive to the particle number: the resonance gets slightly sharper, but its spectral position does not shift. While the behavior in the first case is a manifestation of near-field plasmonic interactions, for the second case the slight narrowing of the resonance could be attributed to an additive effect or related to the emergence of lattice resonances, coupled through far-field scattering/diffractive effects [29].

**Figure 5:**
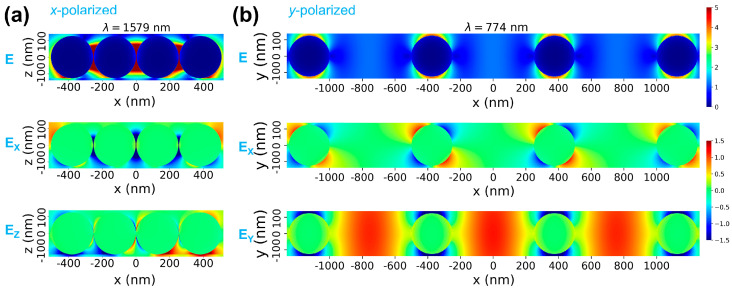
(**a**): Electric field maps under *x*-polarized excitation, for the main red-shifting coupled modes, for quadrumers with *R* = 125 nm and *d* = 3 nm. (**b**): Electric fields under *y*-polarized excitation, for quadrumers with *R* = 125 nm and *d* = 500 nm, at the wavelength of the blue-shifting resonance.

In order to gain more understanding of the coupled plasmon modes discussed above, the electric field distribution was analyzed in relevant cross-sections through the nanosphere oligomers. Figure 5 presents the electric field magnitude *E* together with the *E_X_* and *E_Z_* components, for quadrumers of two configurations: (i) interparticle distance *d* = 3 nm for *x*-polarized incidence, and (ii) *d* = 500 nm for *y*-polarized incidence, at the wavelengths corresponding to the dipole modes for *R* = 125 nm. In the first case, the electric fields exhibit a distribution that is very similar to the one observed also for smaller spheres (Figure S8 of the Supplementary Materials), indicating the same type of coupling occurs in the oligomers. This is consistent with the fact that the resonances for all sphere sizes are red-shifting ones.

For comparing these electric field maps to those for dimers and trimers, and relative to the single sphere case, please see Figures S5–S7 of the Supplementary Materials. On panel (b) of Figure 5, on the other hand, presenting the electric fields associated with the blue-shifting resonance, a few observations are to be noted, as follows: (i) all particles in the quadrumer behave in the same way, while for quadrumer cases in panel (a) differences between field distributions around inner and outer spheres are more obvious; the fact that the electric near-fields on the particle surface are almost identical for a particle at the end of the oligomer (which has only one neighbor) and for a particle inside the oligomer (which has two neighbors) suggests that the near-fields are not strongly affected by interparticle distance; (ii) the *E_Y_* electric field component exhibits areas of high intensity at the centers of the large interparticle gaps, also consistent with a long-range coupling effect. (iii) the spatial distribution of the *E_X_* field component presents a quadrupole-type pattern, indicating that an overlap between the stronger dipolar and a quadrupolar mode lies at the origin of the blue-shifting resonance. The enhanced *E_Y_* electric fields far from the particle surface, although less intense than for small interparticle gaps, can prove more efficient for SERS or SEF detection and analysis of large molecules, such as biomolecules or proteins, or even small particles such as nanoplastics, which cannot be physically accommodated by nm-wide interparticle gaps. A similar distribution of electric fields was also observed under *y* polarization for the trimer composed of spheres with *R* = 125 nm and *d* = 500 nm (Figure S9 of the Supplementary Materials). Additional electric field maps are presented in the Supplementary Materials for quadrumers with *R* = 125 nm under *x* polarization for the lower intensity peaks emerging at 784 nm and 920 nm for *d* = 3 nm in Figure S10, and the peak at 684 nm for *d* = 500 nm in Figure S11.

### 3.2. Stability of the Coupled Modes to Size and Gap Variability

**Figure 6:**
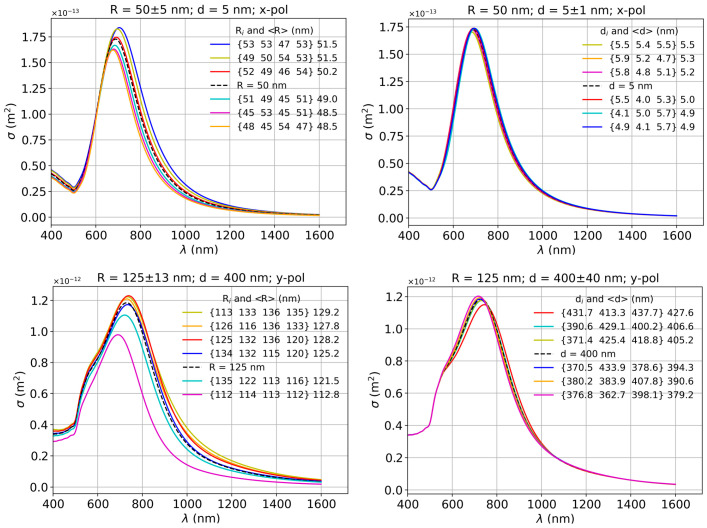
Dependence of the extinction spectrum on nanoparticle size and interparticle gap variations: δ*R* = ±5 nm and δ*d* = ±1 nm, for *R* = 50 nm and *d* = 5 nm; δ*R* = ±13 nm and δ*d* = ±40 nm, for *R* = 125 nm and *d* = 400 nm; in all panels, the legend also shows the average radius of the quadrumers or the average interparticle distance outside the curly brackets. The dashed lines represent the benchmark cases with the nominal size and interparticle gap.

Since in experimental conditions it is very challenging to achieve the same size of the particles or the same interparticle distances, we analyzed some cases where these two parameters may vary by a certain amount. The panels in the left column of Figure 6 show spectra for the variation of the particle size at a constant interparticle distance, specifically for radii in the range of *R* = 50 ± 5 nm and *R* = 125 ± 13 nm, instead of the fixed nominal values of 50 nm and 125 nm, respectively. The right column shows spectra for different interparticle distances in the range *d* = 5 ± 1 nm and *d* = 400 ± 40 nm, instead of the fixed nominal values 5 nm and 400 nm, respectively, for a fixed particle size. As a first observation, the size dispersity seems to have a stronger influence on the intensity and the position of the peak than the interparticle distance dispersity. It is also interesting to note that all the cases, which have an average radius larger than the corresponding nominal *R*, are slightly shifted towards longer wavelengths, while those below the benchmark value are slightly blue-shifted compared to the spectral position of the constant case. Also, the intensity of the peak is higher or lower than for constant *R*, for average radii above and below the benchmark case, respectively.

The right column shows a small effect of the interparticle distance variation for smaller particles and *x* polarization (top panel), while for larger particles and y polarization (bottom panel), we may observe a small shift of the peak towards longer wavelengths for average interparticle distances larger than the benchmark case. Correspondingly, the peaks of the cases with average distances below the benchmark are very slightly shifted towards smaller wavelengths compared to the position of the peak in the constant case. However, in this case, the intensity of the peaks is lower than the benchmark for average interparticle distances larger than the constant *d* case and correspondingly higher for smaller average distances than the constant case. The important consequence of this analysis is that for precise control of the plasmon resonances in such linear oligomers, precise control of the particle size is needed, which is still challenging for the chemical synthesis routes. Small interparticle gaps (few nm) could be controlled by well-defined molecular or polymeric spacers, while large gaps (hundreds of nm) could be controlled through the lithographic definition of the particle placement.

### 3.3. Sensitivity of the Oligomer Systems to Interparticle Distance Change

Next, we analyzed the sensitivity of the oligomer systems to induce interparticle distance change, which in practice can be induced by stretching of the elastomeric substrate. Besides the interest in developing dual-mode SERS/SEF sensing approaches discussed later, this sensitivity is also relevant for developing strain (deformation) sensors [30]. We plotted this quantity (${∆\lambda }_{max}/∆L/L$) as a function of the interparticle gap *d*, where ${∆\lambda }_{max}$ is the difference between wavelength values corresponding to peaks at consecutive gap values, L=2R+d and ∆L is the difference between L values associated with consecutive gap values. In some cases, where the peak associated with a given gap was not conclusive, we did not plot the data point associated with that gap value. It may be observed that the highest sensitivity to deformation (strain) is exhibited by oligomers composed of larger spheres for *x* polarization and for small interparticle distances. The sensitivity decreases as the gap increases in every case for *x* polarization. Additionally, the sensitivity is highest for quadrumers (although for the largest spheres in dipole mode (D), data are shown only for gap values smaller or equal to 20 nm). The sensitivity associated with the quadrupole mode (Q) for *x* polarization shows some increased values at small gaps, but these are still much smaller than for the dipole mode (D), while at larger gap values, the sensitivity essentially goes to zero. For *y*-polarized light, the Q mode again exhibits much smaller values than the D mode, while the latter shows an inversed dependence, with higher sensitivity at large interparticle distances and for a higher number of constituent nanoparticles.

**Figure 7:**
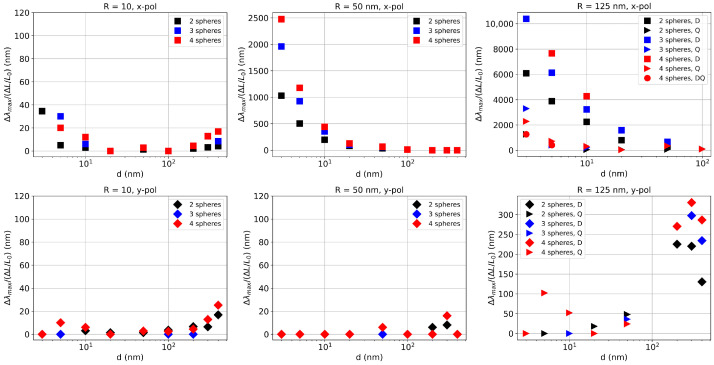
Dependence of the sensitivity of the resonance maximum wavelength on relative elongation (strain).

The most sensitive red-shifting resonance in Figure 7 (*R* = 125, *x*-pol) is however very broad, which is detrimental to developing efficient sensing devices. On the other hand, the red-shifting resonance for (*R* = 50, *x*-pol), besides exhibiting a very high strain dependence, is also narrower and is located in the visible range, making it easier to exploit in practice.

### 3.4. SERS/SEF Applications

**Figure 8:**
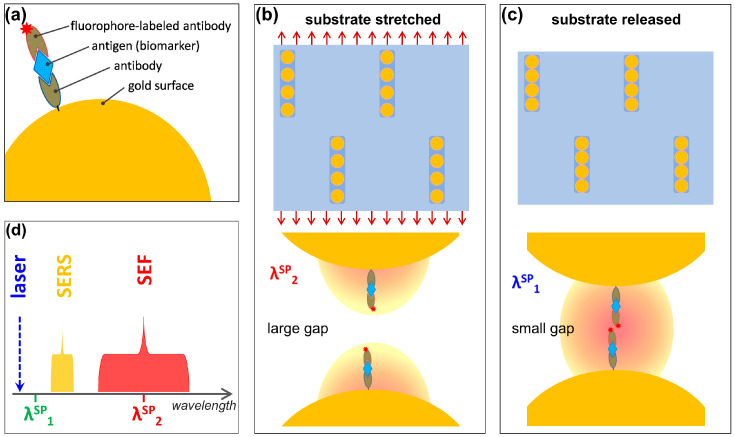
Proposed principle of single-excitation, dual-mode SERS/SEF detection: (**a**) a typical fluorescence-based assay for biomarker detection; (**b**) gold nanosphere linear arrangements on a stretched elastomeric substrate, exhibiting plasmon resonance SP_2_. (**c**) Gold nanospheres on the released substrate, exhibiting plasmon resonance SP_1_. (**d**) Relationship between laser excitation wavelength, SERS and SEF regions, and plasmon resonances SP_1_ and SP_2_.

Finally, we propose a protocol for dual SERS/SEF sensing, based on the resonances that blue-shift with gap decrease. Recently, attempts have been made to combine SEF and SERS in order to exploit a fluorescence read-out for fast imaging and Raman fingerprinting for highly multiplexed molecular identification. In particular, plasmonic nanoparticles encoded with both Raman and fluorescent labels are increasingly gaining attention as bimodal optical probes [31]. However, the two types of labels being spectrally separated, fluorescence (or SEF) was obtained with one excitation wavelength, while SERS was obtained with another laser line [32]. In contrast, we aim to realize plasmonic sensors able to measure both SERS and SEF on the same sample with a single laser excitation. This could be achieved by employing mechanically tunable linear plasmonic oligomers on elastomeric substrates. Figure 8 depicts the principle of the proposed single-excitation, dual-mode SERS/SEF detection: gold nanosphere linear arrangements are first prepared on an elastomeric substrate; upon substrate stretching, this exhibits plasmon resonance SP_2_; the sensor is now exposed to the target analyte, the openings created between the particles favoring the access of the analyte to the interparticle spaces; SEF can be now observed provided the design includes a fluorophore with emission matching SP_2_; next, the stretched substrate is gradually relaxed, and the blue-shifting plasmon resonance will move to SP_1_, causing less overlap between the oligomer resonance and the fluorophore emission, thus a weaker SEF; at the same time, SERS effect can be maximized by resonance SP_1_ approaching the laser excitation and better matching the SERS bands spectral region.

It is worth noting that, while also the red-shifting resonances could be employed for such dual-mode SERS/SEF experiments because of their good sensitivity to strain, this would be achieved for small, few-nm interparticle gaps. For many biosensing applications, this gap would not be sufficient to accommodate the surface functionalization layer and a biomolecule that can be larger than a few nm. For this reason, the blue-shifting resonances of the large sphere oligomers, yielding coupled resonances in the visible range for large interparticle gaps, could prove more advantageous. Despite their much lower field enhancements, these can still have an important impact on emitted/scattered photons by modifying the optical density of states in the emitter/scatterer’s vicinity. Another important practical implication of the possibility of enlarging the openings between the particles is that they can favor the access of the analyte to the interparticle spaces and locations of the highest electromagnetic field enhancements. On many fixed, static plasmonic platforms, it might be the case that the analyte is not able to reach the sites of highest enhancement. Translating these results into a real experimental setting would involve some challenges for fabricating such small nanoparticle oligomers. Although individual oligomers could be studied under an optical microscope, it is more likely that a real sensor or any practical application would consist of many particles. A possibility would be to produce arrays of oligomers at a sufficient distance from one another to avoid interactions between the oligomers, which could possibly be fabricated by colloidal self-assembly onto prepatterned substrates (with grooves, pits, etc.) [33,34,35]. Exploiting DNA origami for creating oligomers would also be a possibility [36,37].

## 4. Conclusions

We have performed a thorough analysis of plasmonic systems consisting of linear oligomers composed of gold nanospheres placed on a dielectric substrate. By using finite-difference time-domain electromagnetic simulations, we obtained the extinction spectra of dimers, trimers, and quadrumers made of gold spheres having 20, 100, or 250 nm diameters, with interparticle gaps in the range 3–500 nm, for incident light polarized parallel or perpendicular to the oligomer axis. The tunability of various coupled plasmon modes by mechanically induced strain (relative substrate elongation) was then evaluated by variation of interparticle gaps. Interestingly, besides the well-known red-shifting dipolar coupling plasmon resonances, we also observed blue-shifting plasmon modes, and then analyzed the distribution of associated electric fields. While the behavior of the red-shifting resonance is due to near-field plasmonic interactions and offers high-field enhancement regions in small gaps, the blue-shifting resonances can be related to long-range interactions, scattering or diffractive effects, and offer smaller enhancements but in larger gaps. Based on these findings, an approach for dual-mode SERS/SEF sensing using a single excitation wavelength could be developed by mechanically tuning the coupled plasmon resonances either to the spectral range of SERS or SEF. The oligomers consisting of large (250 nm) nanospheres, exhibiting blue-shifting modes, offer more versatility in real-time mechanical control of the optical response of such linear oligomers, facilitating the future design of new optical (bio)sensing protocols.

## Data Availability

The data presented in this study are available on request from the corresponding author.

## Supplementary Materials

The following supporting information can be downloaded at: https://www.mdpi.com/article/10.3390/bios15030181/s1, Figure S1: (left) Illustration of the simulation setup including an additional fine mesh in the interparticle region. (right) Simulation result for the two cases: 1 nm mesh over the entire particles, and a 1 nm mesh over the particles with a supplementary 0.2 nm mesh in the gap region, for the case of 4 spheres with *R* = 50 nm, and *d* = 3 nm, x-polarization. Figure S2: Extinction spectra for dimers, trimers, and quadrumers made of gold nanospheres (*R* = 10 nm) as a function of interparticle distance (*d* = 3 to 500 nm), for the x and y polarizations of the incident light. Figure S3: Extinction spectra for dimers, trimers, and quadrumers made of gold nanospheres (*R* = 50 nm) as a function of interparticle distance (*d* = 3 to 500 nm), for the x and y polarizations of the incident light. Figure S4: Unpolarized extinction spectra obtained by averaging the corresponding x- and y-polarized responses. Figure S5: Electric fields near the single nanoparticle: (left) *R* = 10 nm; (center) *R* = 50 nm; (right) *R* = 125 nm. Figure S6: Electric fields near nanoparticle dimers: *d* = 3 nm; (left) *R* = 10 nm; (center) *R* = 50 nm; (right) *R* = 125 nm. Figure S7: Electric fields near nanoparticle trimers: *d* = 3 nm; (left) *R* = 10 nm; (center) *R* = 50 nm; (right) *R* = 125 nm. Figure S8: Electric fields near nanoparticle quadrumers: *d* = 3 nm; (left) *R* = 10 nm; (center) *R* = 50 nm; (right) *R* = 125 nm. Figure S9: Electric fields near nanoparticle trimers: *R* = 125 nm, *d* = 500 nm. Figure S10: Electric fields near nanoparticle quadrumers: *R* = 125 nm, *d* = 3 nm. Figure S11: Electric fields near nanoparticle quadrumers: *R* = 125 nm, *d* = 500 nm.
