# Supplementary material for: Dynamic Plasmonic Coupling in Gold Nanosphere Oligomers: Mechanically Tuned Red and Blue Shifts for SERS/SEF
Source: Biosensors (Basel). 2025 Mar 13;15(3):181. doi: 10.3390/bios15030181 (PMC11940356; doi:10.3390/bios15030181)
Supplement: Supplementary file 1 [file biosensors-15-00181-s001.zip › biosensors-3411481-supplementary.pdf]

SUPPLEMENTARY MATERIAL

Dynamic Plasmonic Coupling in Gold Nanosphere Oligomers: Mechanically Tuned Red and Blue Shifts for SERS/SEF

István Tóth, Cosmin Farcău

National Institute for Research and Development of Isotopic and Molecular Technologies, 67-103 Donat, 400293 Cluj-Napoca, Romania

*email: cfarcau@itim-cj.ro

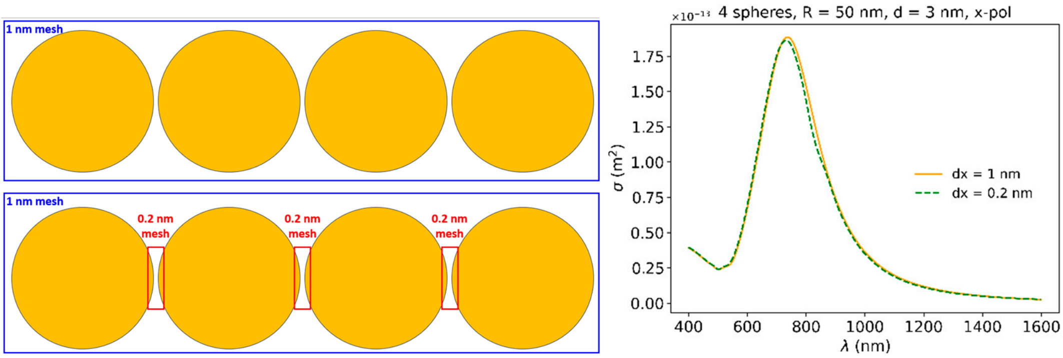

Figure S1. (left) Illustration of the simulation setup including an additional fine mesh in the interparticle region. (right) Simulation result for the two cases: 1 nm mesh over the entire particles, and a 1 nm mesh over the particles with a supplementary 0.2 nm mesh in the gap region, for the case of 4 spheres with R=50 nm, and d=3 nm, *x*-polarization.

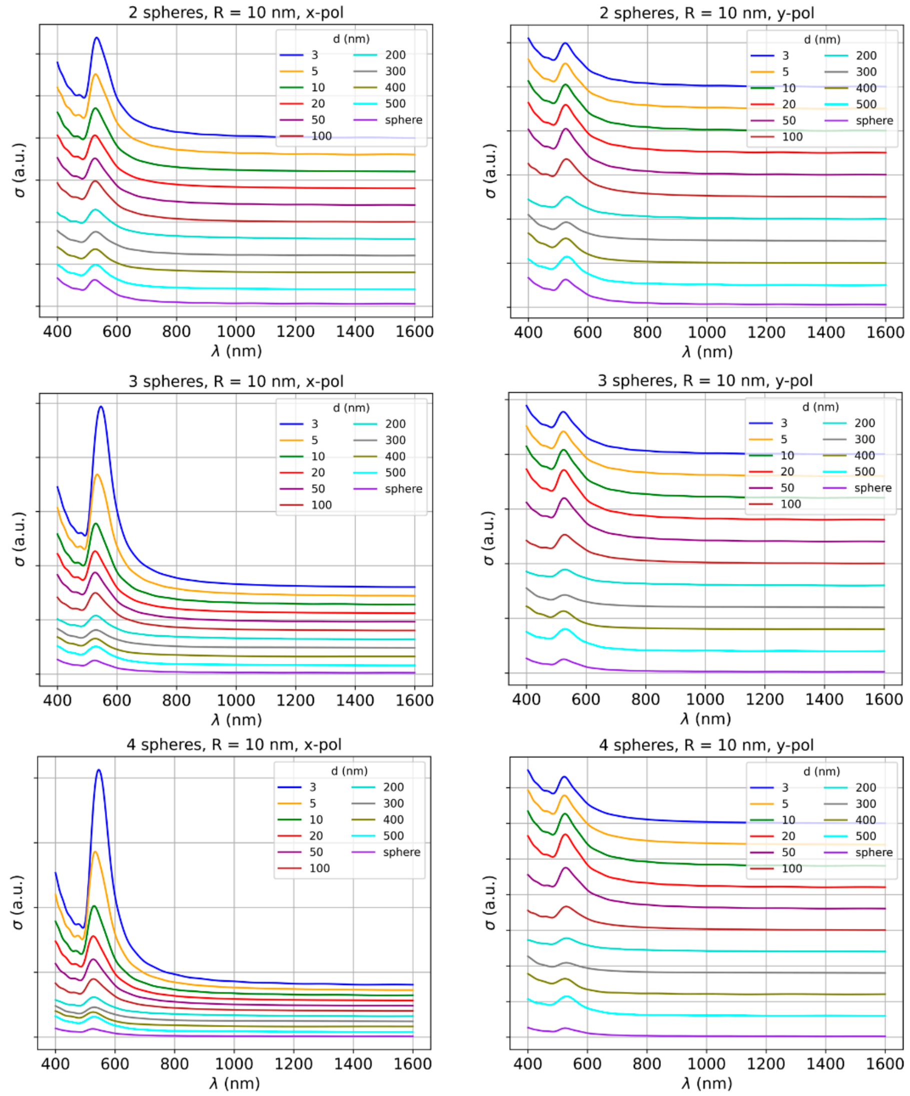

Figure S2. Extinction spectra for dimers, trimers, and quadrumers made of gold nanospheres (R = 10 nm) as a function of interparticle distance (d = 3 to 500 nm), for the x and y polarizations of the incident light.

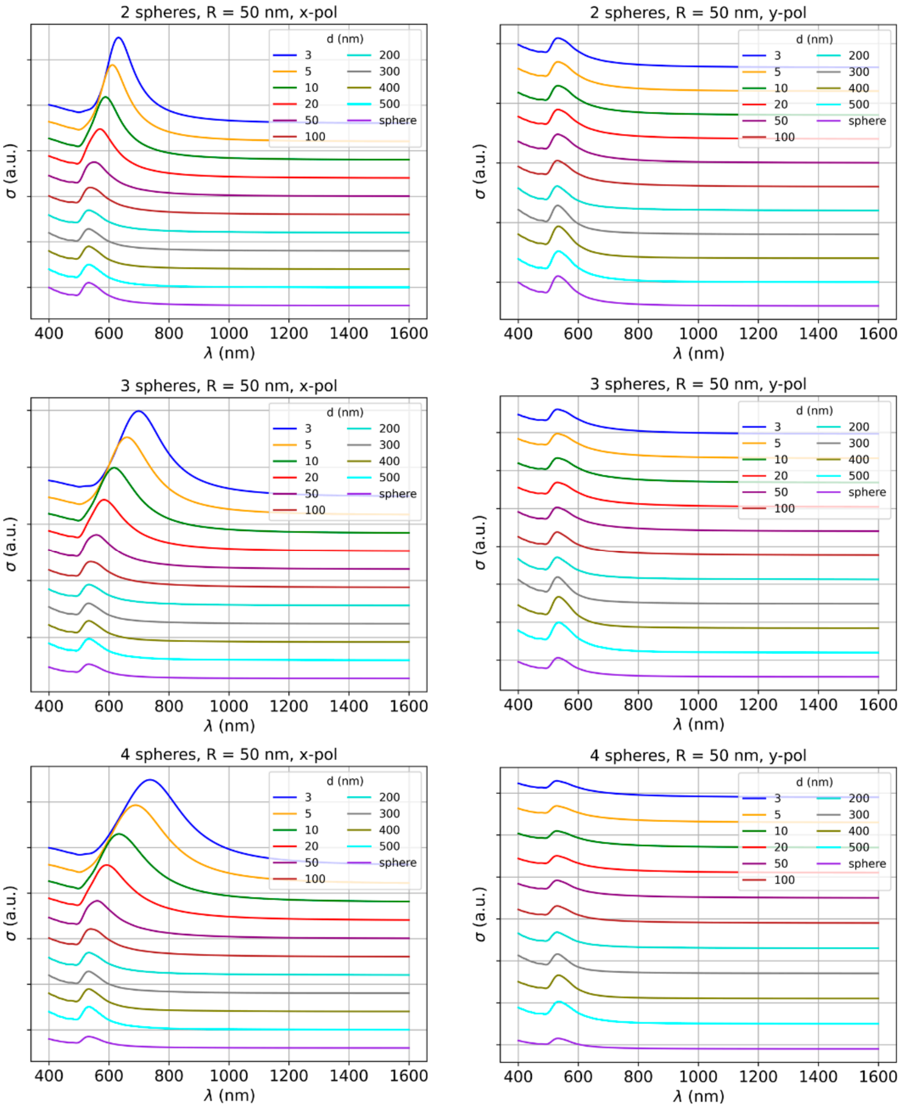

Figure S3. Extinction spectra for dimers, trimers, and quadrumers made of gold nanospheres (R = 50 nm) as a function of interparticle distance (d = 3 to 500 nm), for the x and y polarizations of the incident light.

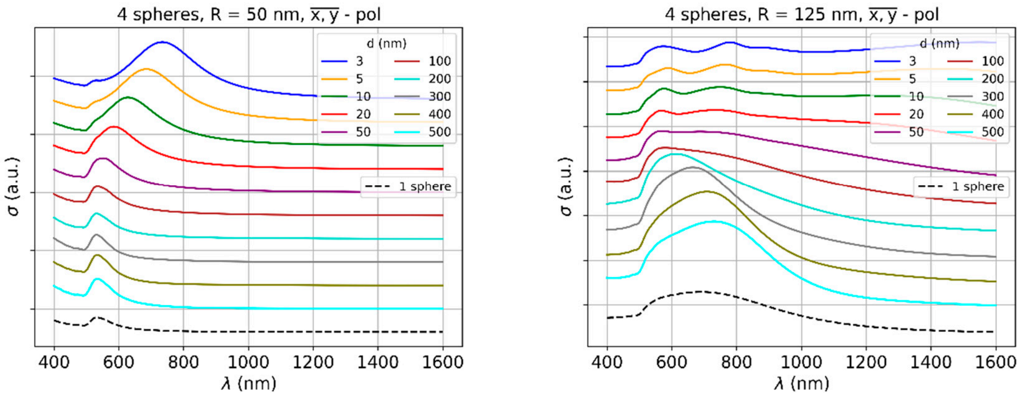

Figure S4. Unpolarized extinction spectra obtained by averaging the corresponding *x*- and *y*-polarized responses.

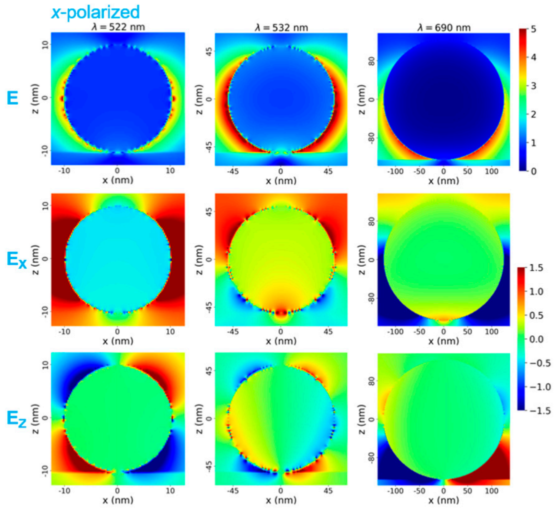

Figure S5. Electric fields near the single nanoparticle: (left) *R* = 10 nm; (center) *R* = 50 nm; (right) *R* = 125 nm.

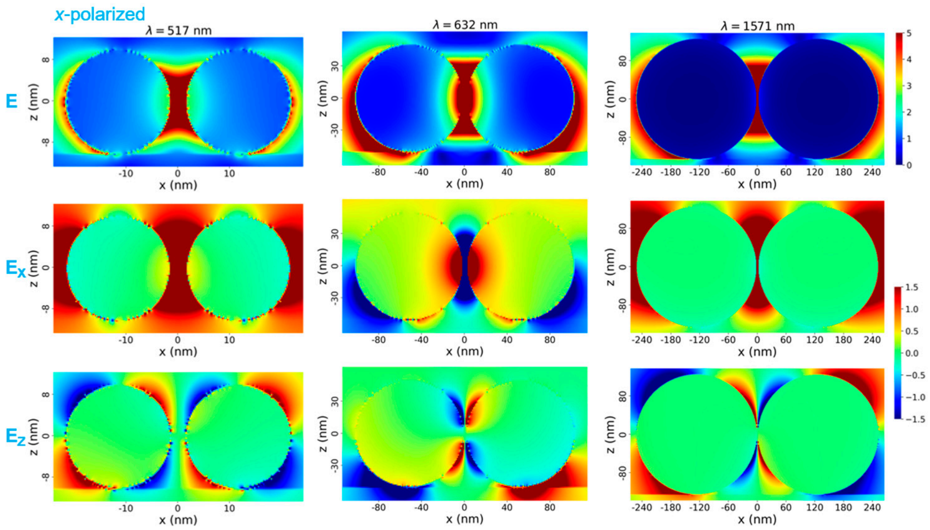

Figure S6. Electric fields near nanoparticle dimers: d = 3 nm; (left) R = 10 nm; (center) R = 50 nm; (right) R = 125 nm.

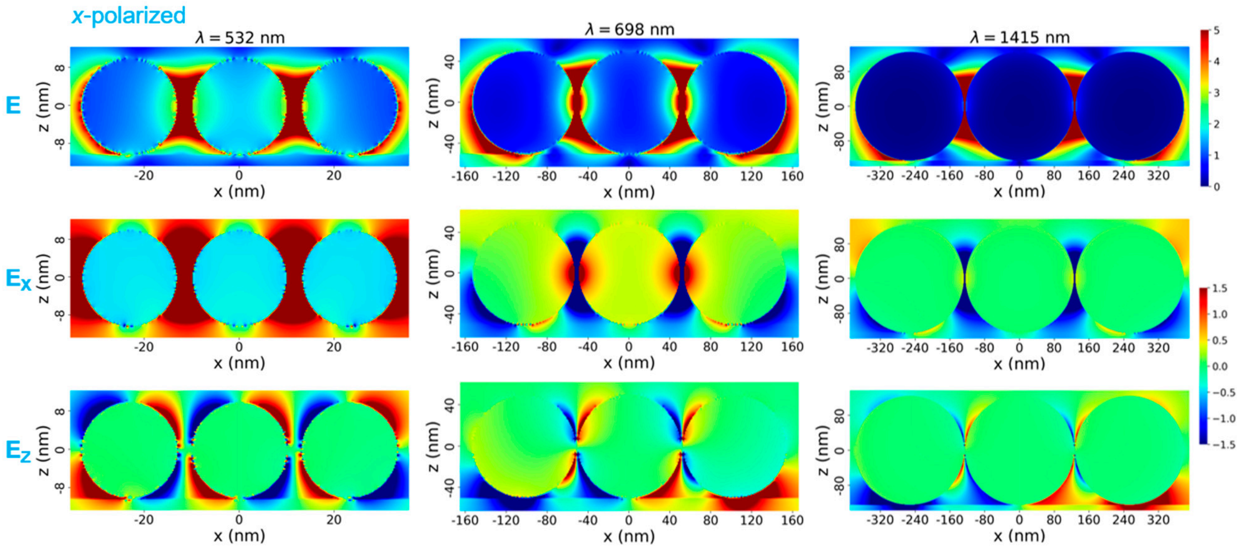

Figure S7. Electric fields near nanoparticle trimers: d = 3 nm; (left) R = 10 nm; (center) R = 50 nm; (right) R = 125 nm.

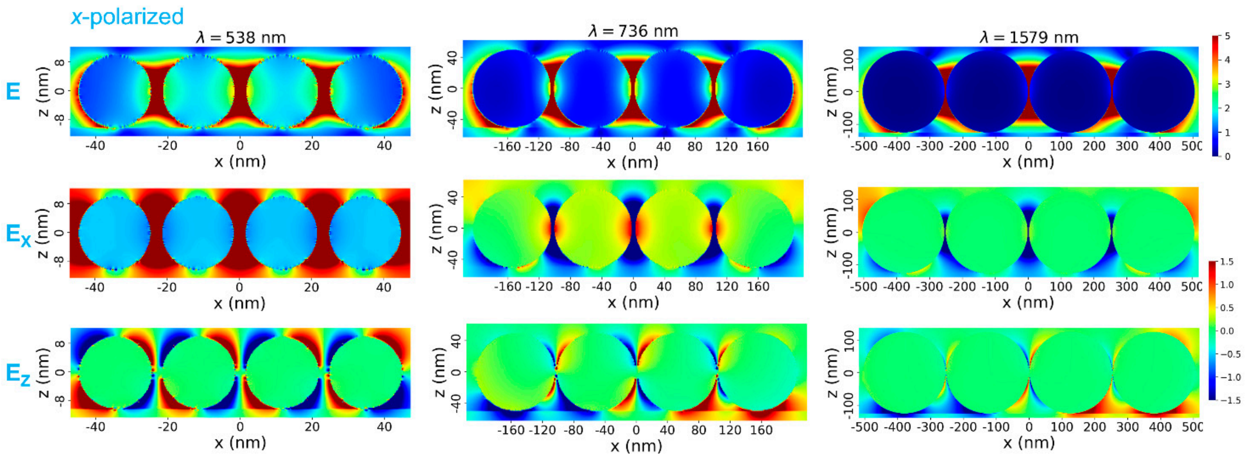

Figure S8. Electric fields near nanoparticle quadrumers: d = 3 nm; (left) R = 10 nm; (center) R = 50 nm; (right) R = 125 nm.

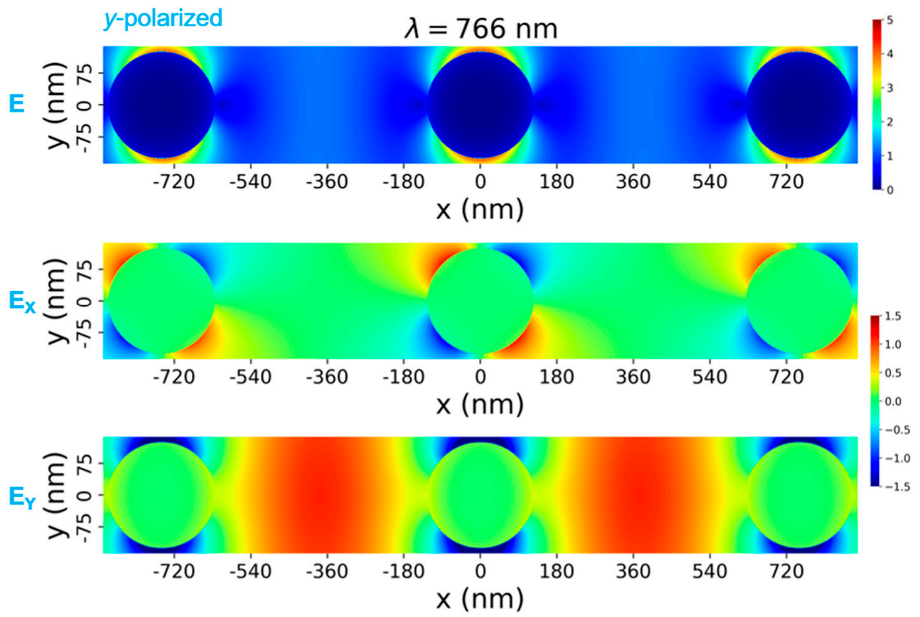

Figure S9. Electric fields near nanoparticle trimers: R = 125 nm, d = 500 nm.

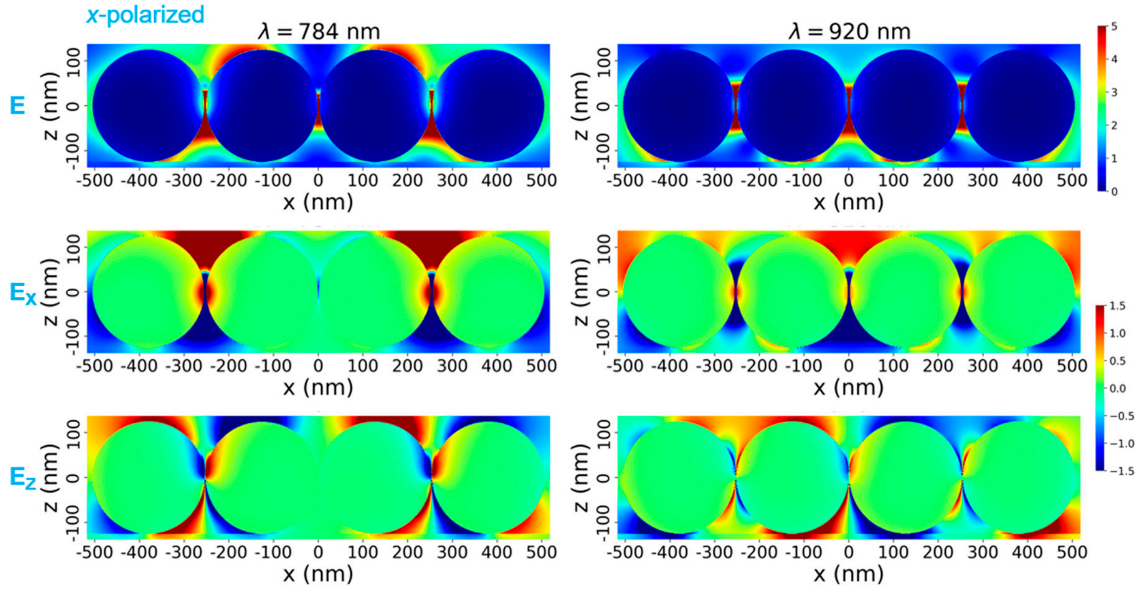

Figure S10. Electric fields near nanoparticle quadrumers: R = 125 nm, d = 3 nm.

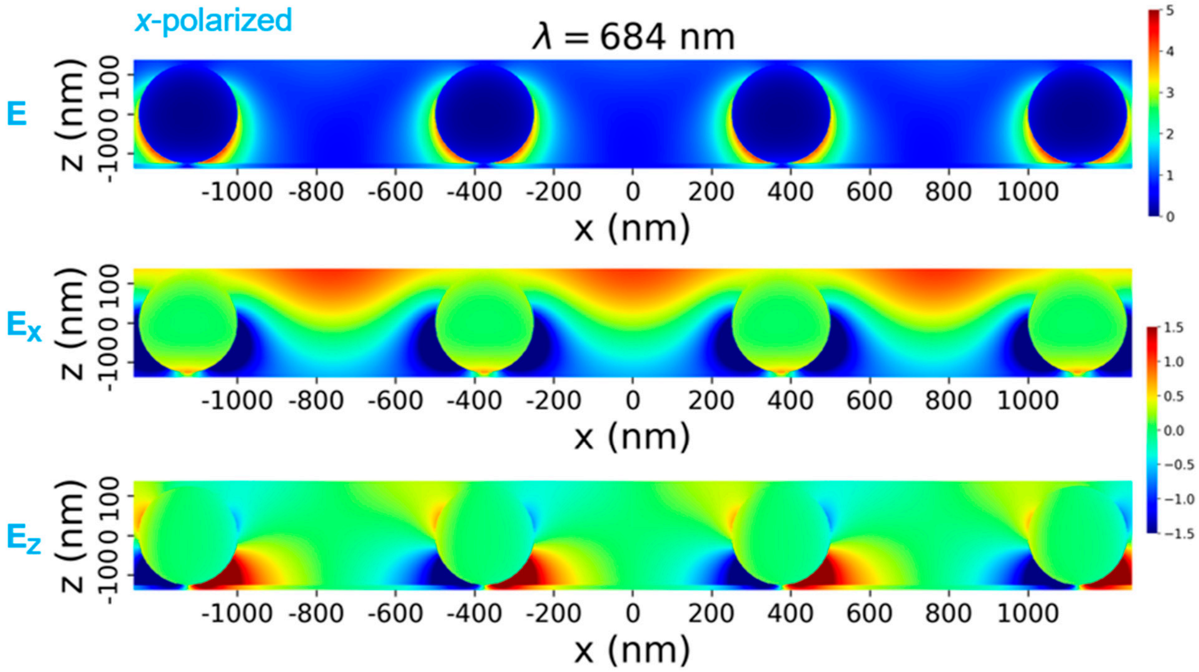

Figure S11. Electric fields near nanoparticle quadrumers: R = 125 nm, d = 500 nm.
